# Cidofovir is active against human papillomavirus positive and negative head and neck and cervical tumor cells by causing DNA damage as one of its working mechanisms

**DOI:** 10.18632/oncotarget.10100

**Published:** 2016-06-16

**Authors:** Barbara Mertens, Tatiane Nogueira, Ruzena Stranska, Lieve Naesens, Graciela Andrei, Robert Snoeck

**Affiliations:** ^1^ Rega Institute for Medical Research, KU Leuven, 3000 Leuven, Belgium

**Keywords:** human papillomavirus, cidofovir, DNA damage, incorporation, antiproliferative effects

## Abstract

Human papillomavirus (HPV) causes cervical cancer and a large fraction of head and neck squamous cell carcinomas (HNSCC). Cidofovir (CDV) proved efficacious in the treatment of several HPV-induced benign and malignant hyper proliferations. To provide a better insight into how CDV selectively eradicates transformed cells, HPV^+^ and HPV^−^ cervical carcinoma and HNSCC cell lines were compared to normal cells for antiproliferative effects, CDV metabolism, drug incorporation into cellular DNA, and DNA damage. Incorporation of CDV into cellular DNA was higher in tumor cells than in normal cells and correlated with CDV antiproliferative effects, which were independent of HPV status. Increase in phospho-ATM levels was detected following CDV exposure and higher levels of γ-H2AX (a quantitative marker of double-strand breaks) were measured in tumor cells compared to normal cells. A correlation between DNA damage and CDV incorporation into DNA was found but not between DNA damage and CDV antiproliferative effects. These data indicate that CDV antiproliferative effects result from incorporation of the drug into DNA causing DNA damage. However, the anti-tumor effects of CDV cannot be exclusively ascribed to DNA damage. Furthermore, CDV can be considered a promising broad spectrum anti-cancer agent, not restricted to HPV^+^ lesions.

## INTRODUCTION

Human papillomaviruses (HPVs) are double-stranded DNA viruses with a small genome of about 8 kbp. HPVs can induce benign (low-risk types) and malignant (high-risk types) lesions. High-risk HPVs are involved in almost all cases of cervical carcinoma, a number of other anogenital cancers and an increasing amount of head and neck squamous cell carcinomas (HNSCC) [1–3].

HPVs have a tropism for epithelial cells and cancer progression is associated with a persistent infection caused by a high-risk HPV type (such as HPV type 16, 18, 31, 33 and 45). When an HPV infection evolves into cancer, the episomal viral DNA frequently becomes integrated into the host-cell DNA resulting in a loss of viral gene expression, except for the genes encoding the HPV E6 and E7 oncoproteins [4]. These two oncoproteins are responsible for cell transformation and immortalization [5]. Among several functions, E6 causes degradation of the tumor suppressor p53 [6] while one of the most important functions of E7 is the association with the retinoblastoma family of proteins (pRB), abrogating DNA repair and maintenance of genomic integrity. In contrast to normal cells, tumor cells are often unable to repair DNA damage because of defects in their DNA damage response mechanisms [7]. Upon double-strand breaks (DSBs), the ataxia telangiectasia mutated (ATM) protein is activated by phosphorylation of a serine at position 1981 which causes monomerization and transport of ATM to the sites of DNA damage. The stabilization of ATM at DNA damage sites is required for a proper DNA damage response [8]. Phospho-ATM can be dephosphorylated by protein phosphatase 2 (PPA2) and by wild-type p53-induced phosphatase 1 (Wip1). These phosphatases prevent illicit activation of the DNA damage response in the absence of damage and allow rapid cessation of the signal once DNA damage is repaired [9]. Activated ATM is responsible for the phosphorylation of the H2A histone family member X (H2AX) protein at serine 139 resulting in γ-H2AX (phosphorylated-H2AX) foci at the sites of DNA damage. Phosphorylation of H2AX was shown to be important to recruit repair proteins to DNA damage sites and γ-H2AX is considered a sensitive and quantitative indicator of DSBs [10].

Although there are effective prophylactic vaccines available for HPV, treatment of HPV infections remains a challenge [11]. HPV vaccination is not applied worldwide and generally only adolescent women are vaccinated, leaving men, relatively older women and non-vaccinated populations unprotected [12]. Current treatment of early stage cervical cancer consists of surgical removal of the cervical cone (conization). Hysterectomy with radio- and/or chemotherapy is the treatment of choice for women with invasive cervical cancer [13]. HNSCC patients are currently treated by either surgery or a combination of chemo- and radiotherapy [14]. These interventions are generally effective although a substantial morbidity and disease recurrence are described [15, 16].

Cidofovir [(CDV), [(S)-1-[3-hydroxy-2-(phosphonomethoxy)propyl]cytosine], HPMPC, Vistide^®^] is an acyclic nucleoside phosphonate (ANP) known for its broad-spectrum antiviral activity against DNA viruses. CDV was approved by the FDA for the treatment of cytomegalovirus (CMV) retinitis in AIDS patients [17]. CDV also proved to be efficacious in patients suffering from HPV-associated diseases including severe laryngeal papillomatosis, anogenital papillomavirus infections and cervical intraepithelial neoplasia stages 2+ and 3 [18–22]. CDV was shown to have antiproliferative properties against HPV^+^ cervical carcinoma and HPV^−^ transformed cell lines [23]. Moreover, CDV has not only been demonstrated to improve the pathology caused by the growth of HPV^+^ xenografts in athymic nude mice [24], but also against a number of HPV^−^ malignancies *in vivo* like glioblastoma, hemangiosarcoma and nasopharyngeal carcinoma [25–28].

CDV requires two phosphorylation steps in order to be active. The first phosphorylation is catalyzed by the cytosolic UMP-CMP kinase, producing CDV-monophosphate (CDVp) which is then phosphorylated by a nucleoside diphosphate kinase, pyruvate kinase or creatine kinase to the diphosphate form (CDVpp). The intracellular depot form of CDV, cidofovir monophosphocholine (CDVp-choline) is formed by choline-phosphate cytidylyltransferase [29–31]. CDVpp is the active metabolite and can be incorporated into DNA instead of the natural substrate dCTP [17].

The antiproliferative effects of CDV against HPV^+^ cervical cancer cell lines were reported for the first time in 1998 [23]. In contrast to other chemotherapeutic agents, inhibition of cell growth by CDV increased in function of time [23]. Today, the molecular mechanisms underlying the selectivity of CDV for transformed cells are not completely understood. To investigate the selective effects of CDV for tumor cells compared to normal cells, our group performed a comprehensive analysis of gene expression profiling by means of microarray in cervical cancer cells [SiHa (HPV16^+^) and HeLa (HPV18^+^)], immortalized keratinocytes (HaCaT) and primary human keratinocytes (PHKs), exposed or not to CDV. Functional classification of differentially expressed genes, using Ingenuity Pathway Analysis software, was performed to identify functional categories and molecular pathways changed following CDV exposure in transformed cells *versus* normal cells. Cell cycle regulation and DSB repair mechanisms, such as ATM signaling and DSB repair by homologous recombination were found to be activated in CDV-exposed PHKs but not in transformed cells. These data pointed to the generation of DSBs following CDV exposure [32]. Furthermore, previous results revealed that CDV selectivity for HPV transformed cells may be based on differences in replication rates and on CDV incorporation into genomic DNA between cancer cells (SiHa, HeLa and HaCaT) and normal cells (PHKs) [32]. Here we have demonstrated at the protein level that CDV induces DSBs in different tumor cell types. Induction of DNA damage by CDV was compared with antiproliferative effects and drug incorporation into DNA in our studies using both high-risk HPV^+^ and HPV^−^ HNSCC and cervical carcinoma cell lines as well as normal cells. We demonstrate here a correlation between DNA incorporation of CDV and DNA damage and between CDV incorporation and antiproliferative effects but not between DNA damage and CDV antiproliferative effects. Our findings also support the applicability of CDV as a broad spectrum antitumor agent against both HPV^+^ and HPV^−^ tumors.

## RESULTS

### Antiproliferative effects of CDV on HPV^+^ and HPV^−^ tumor cells and normal cells

The antiproliferative effects of CDV were evaluated in HPV^+^ and HPV^−^ transformed cells as well as normal cells. Before performing these experiments, the HPV positivity and negativity of all cell lines was confirmed by means of PCR with specific primers for the detection of HPV16, HPV18 and HPV33. All cells were tested for the three HPV types and the HPV16 positivity of SiHa, Caski, SCC-147, UM-SCC-47, UD-SCC-2 and UM-SCC-104 was confirmed. HeLa cells proved to be HPV18^+^ and CK1 and UT-SCC-45 were HPV33^+^. The other cell lines (i.e. C33A, SCC-9, SCC-4, SCC-120, UM-SCC-38 and HaCaT) and the normal human diploid cells (i.e. HEL, PHK and PET) were negative for HPV16, HPV18 or HPV33.

**Figure 1:**
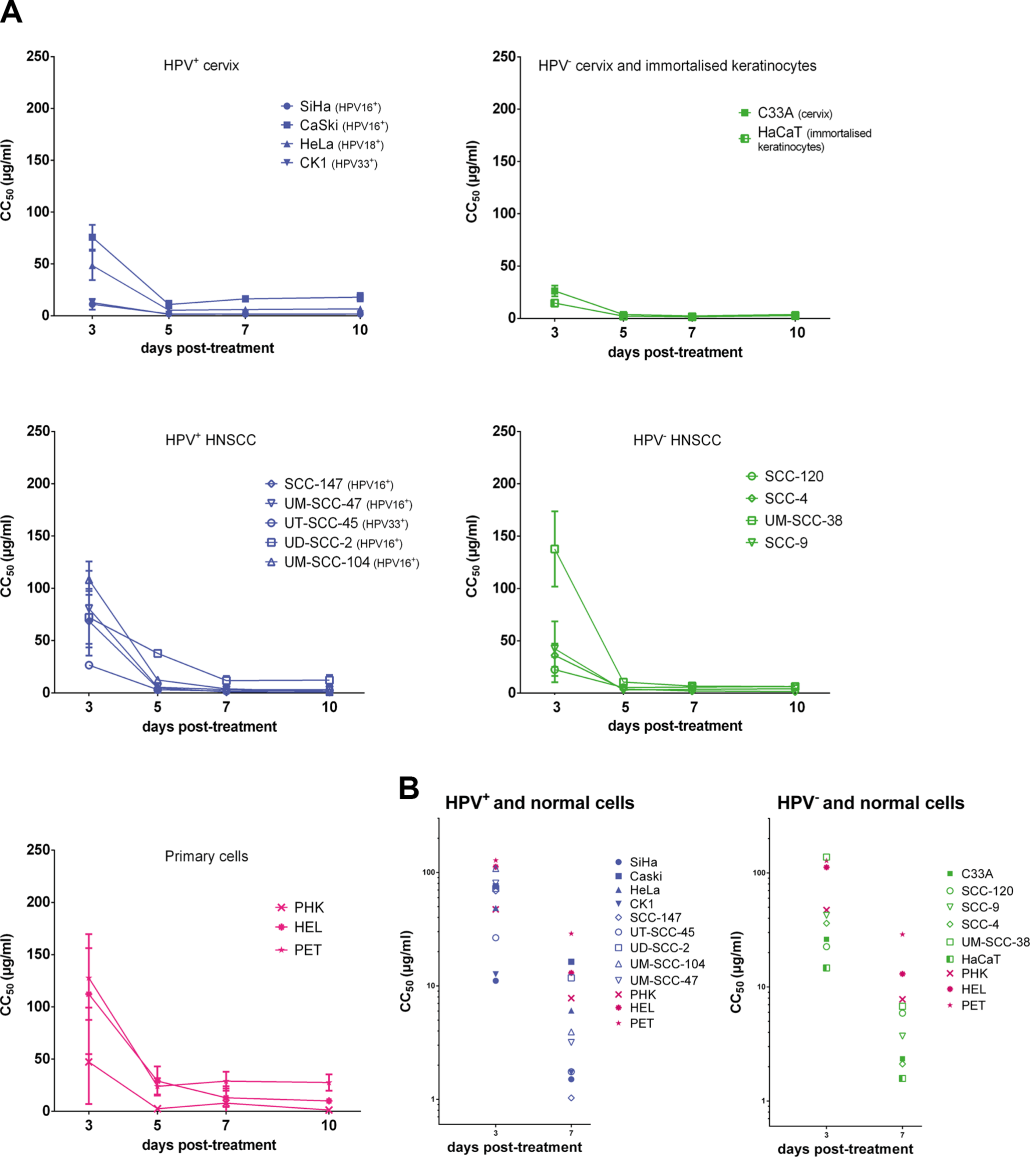
Antiproliferative effects of CDV CC_50_ values in function of days post treatment. Cells seeded in a 96 well plate were treated after 24 h with dilutions of CDV ranging from 0.05 μg/ml to 200 μg/ml. At 3, 5, 7 and 10 days post treatment, cells were counted with a Coulter counter to calculate CC_50_ values. The CC_50_ value is the concentration of CDV needed to inhibit cell growth by 50%. HPV^+^ cell lines are indicated in blue, HPV^−^ in green and the normal cells in pink. Cervical cancer cell lines are indicated by closed symbols and HNSCC by open symbols. Values are shown as mean of minimum 3 experiments per cell line and SEM. (**A**) CC_50_ values in function of day 3, 5, 7 and 10 post-treatment. (**B**) CC_50_ values in function of day 3 and 7 post-treatment.

The antiproliferative effects of CDV on the different cells were measured at 3, 5, 7 and 10 days post-exposure to CDV (Figure 1A). First, the CC_50_ values at 3 days post-treatment were compared for the different cell lines (Figure 1B). Lower CC_50_ values at 3 days post-treatment were observed for most of the transformed cell lines in comparison with normal cells, showing the selectivity of CDV for tumor cells. SiHa, CK1, HaCaT and SCC-120 were significantly more sensitive to CDV after 3 days of treatment than PHK, HEL and PET cells. Also HeLa cells, SCC-147, UT-SCC-45, SCC-4, SCC-9 and C33A showed lower CC_50_ values than PET and HEL cells, but they were not significantly different from PHKs. UD-SCC-2, UM-SCC-47 and Caski showed a difference in CC_50_'s with PET cells 3 days post-treatment but not with the two other normal cells. UM-SCC-104 and UM-SCC-38 had a sensitivity to CDV comparable to that of normal cells.

Our results showed that the antiproliferative activity of CDV significantly increased over time from day 3 to day 10 post-treatment in all the cell lines tested, except for Caski and SCC-120, where the increase was not significant (Figure 1A).

After 7 days of treatment, CDV inhibited the growth of most of the cancer cell lines (SiHa, CK1, SCC-147, UM-SCC-47, UT-SCC-45, UM-SCC-104, UM-SCC-38, C33A, HaCaT, SCC-120, SCC-4, SCC-9) in comparison with HEL and PET cells (Figure 1B). However, no significant differences in CC_50_ values between these tumor cell lines and PHKs were observed. The proliferation of HeLa and UD-SCC-2 cells was significantly reduced by CDV at 7 days post-treatment compared to PET cells, but not compared to PHKs and HEL cells. The CC_50_ values of Caski were not different from those of normal cells at 7 days post-treatment.

The doubling time (DT) of the different cells was calculated to evaluate the *in vitro* growth rate of the cell lines in the absence of CDV. As cancer cells are by definition fast proliferating cells, the tumor cell lines showed shorter doubling time than normal cells (Table 1). A significant difference in doubling time was observed between PET cells and all the cancer cells while PHK showed a significantly longer doubling time than the tumor cells except for SCC-147, UM-SCC-104 and UD-SCC-2. Differences in doubling time between tumor cells and HEL cells were observed for most of the tumor cells except for SCC-147, UM-SCC-104, UT-SCC-45, UD-SCC-2 and SCC-4.

**Table 1:** Doubling time of the cells

Cells (HPV^+^)	DT ± SEM	Cells (HPV^−^)	DT ± SEM	Primary cells	DT ± SEM
**SiHa**	21.98 ± 1.08	**C33A**	27.69 ± 4.32	**PHK**	60.52 ± 5.34
**Caski**	24.92 ± 1.26	**HaCaT**	24.54 ± 2.65	**HEL**	52.04 ± 6.73
**HeLa**	24.06 ± 1.02	**SCC-120**	27.76 ± 2.15	**PET**	110.47 ± 11.21
**CK1**	25.79 ± 1.10	**SCC-4**	40.71 ± 2.11		
**SCC-147**	43.42 ± 6.12	**SCC-9**	33.89 ± 2.08		
**UM-SCC-47**	37.63 ± 1.47	**UM-SCC-38**	35.54 ± 3.11		
**UM-SCC-104**	62.07 ± 5.43				
**UT-SCC-45**	38.52 ± 3.95				
**UD-SCC-2**	43.31 ± 5.55				

Mean doubling time (DT), in hours, and SEM of the cell lines. At least 3 experiments were performed per cell line. Untreated cells were seeded in 96 well plates and counted at the same time points as for the antiproliferative experiments. DT was calculated with the formula: DT = (t_2_–t_1_)/(log_2_N_2_−log_2_N_1_), where t_1_ and t_2_ are the times (hours) at which the cells were counted, and N_1_ and N_2_ are the cell numbers at times t_1_ and t_2_.

**Figure 2:**
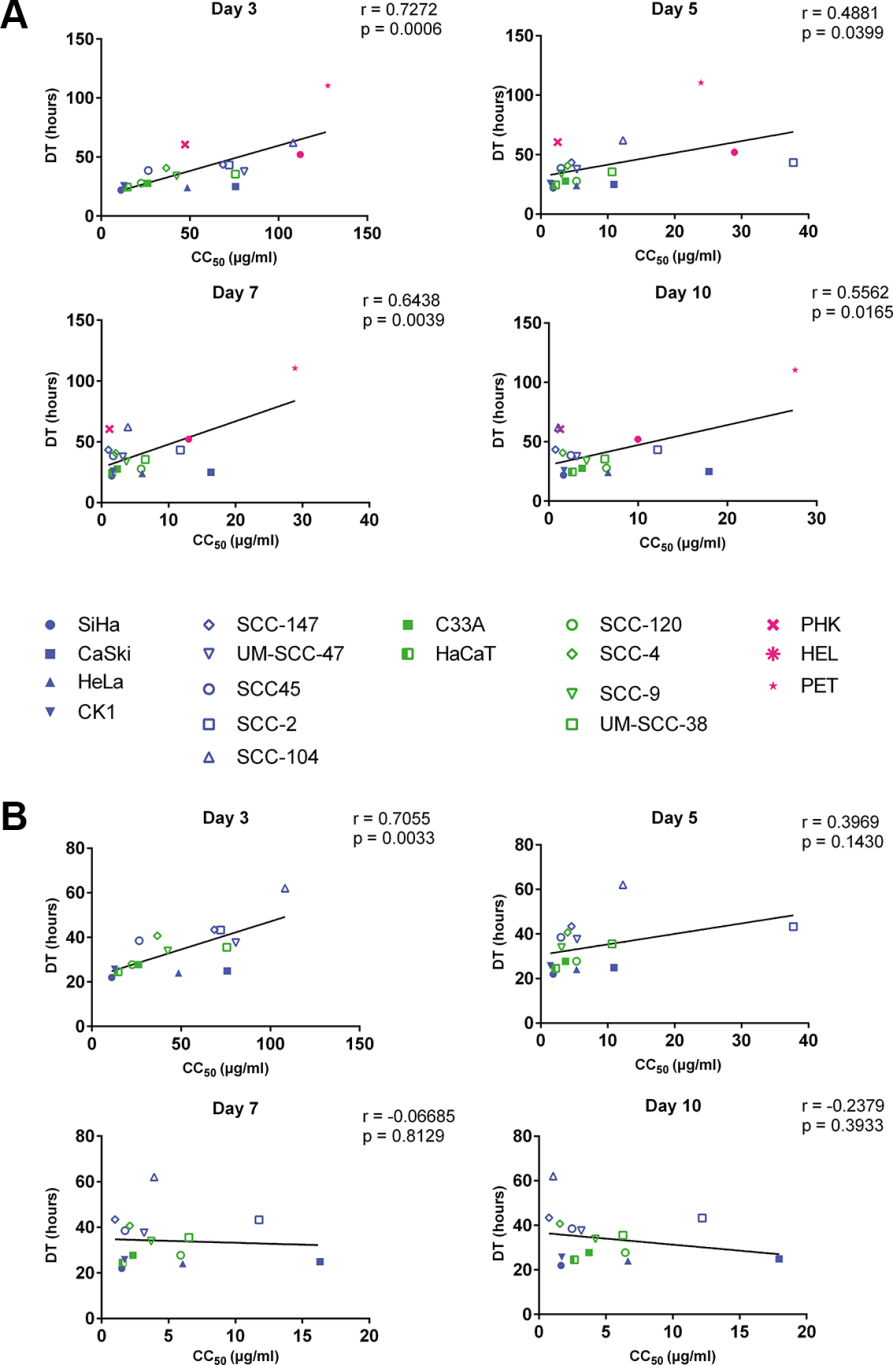
Correlation between doubling time and antiproliferative effects of CDV at days 3, 5, 7 and 10 Pearson correlation was made between DT and CC_50_ values. HPV^+^ cell lines are indicated in blue, HPV^−^ in green and the normal cells in pink. Cervical cancer cell lines are indicated by closed symbols and HNSCC by open symbols. (**A**) All the cell lines. (**B**) Tumor cell lines with exclusion of the normal cells.

A correlation between doubling time and CC_50_ values of all tested cell types, including normal cells, was observed at day 3 (*p* < 0.001), 5 (*p* < 0.05), 7 (*p* < 0.01) and 10 (*p* < 0.05) post-treatment, indicating that the growth of slow proliferating cells is less inhibited by CDV than that of fast proliferating cells (Figure 2A). When the normal cells were excluded from the analysis, only a significant correlation between CC_50_ values and doubling time was observed after 3 days of treatment (*p* < 0.01) but not at later time points (Figure 2B), indicating that the sensitivity of tumor cells to CDV does not depend on their growth rate at the later time points.

Based on these results, representative cell lines were selected for further studies on CDV metabolism, drug incorporation into DNA and induction of DNA damage in order to investigate the antiproliferative mechanisms of CDV. Cervical cancer cell lines were selected to have a representative HPV16^+^ and HPV18^+^ cell line (SiHa and HeLa, respectively) and an HPV^−^ cell line (C33A). One HPV16^+^ (SCC-147) and one HPV^−^ (SCC-120) HNSCC were also selected as well as the spontaneously transformed keratinocytes (HaCaT). The normal cells (PHKs, PET and HEL) were included as controls.

### CDV metabolism and incorporation into DNA

CDV metabolism and incorporation into DNA were examined following incubation of the different cell types with radiolabeled compound. The CDV metabolites in the methanol soluble fraction were separated by HPLC analysis (Table 2). In all cell types, non-metabolized CDV and its depot form (i.e. CDVp-choline) were detected at higher levels than the two phosphorylated metabolites (i.e. CDVp and CDVpp). CDVp was less abundant than CDVpp in all the cell types except for PET cells. It needs to be mentioned that the amount of CDVpp in the methanol soluble fraction represents the free metabolite and therefore, does not include CDV incorporated into cellular DNA.

**Table 2:** CDV metabolites and CDV incorporation into genomic DNA

	SiHa	HeLa	SCC-147	SCC-120	HaCaT	C33A	PHK	PET	HEL
[pmol/10e6 cells]									
Methanol-soluble fraction									
CDVp-choline	155.84 ± 12.03	94.42 ± 5.89	154.79 ± 8.23	183.84 ± 22.56	54.03 ± 9.21	111.21 ± 27.99	983.77 ± 253.66	573.34 ± 147.20	170.53 ± 31.87
CDV	200.35 ± 57.03	137.08 ± 54.41	234.96 ± 80.03	153.94 ± 33.44	63.58 ± 14.04	135.63 ± 19.07	421.12 ± 56.83	348.27 ± 118.32	132.52 ± 32.60
CDVp	52.22 ± 5.26	23.84 ± 4.67	39.4 ± 9.89	39.77 ± 5.25	22.58 ± 13.35	40.02 ± 7.43	268.48 ± 174.84	120.72 ± 17.37	54.88 ± 6.49
CDVpp	74.98 ± 8.46	63.83 ± 26.50	90.09 ± 10.43	78.16 ± 11.83	28.53 ± 5.5	61.07 ± 17.92	409.61 ± 85.99	118.40 ± 29.54	124.06 ± 24.85
sum of metaboites	483.39 ± 29.57	319.17 ± 30.49	519,2 ± 40.86	455.71 ± 21.18	168.72 ± 11.07	347.93 ± 19.51	2082.98 ± 162.43	1160.73 ± 95.97	481.99 ± 26.16
CDV incorporation	133.02 ± 47.37	67.91 ± 23.05	76.33 ± 22.96	79.01 ± 22.94	91.35 ± 24.38	74.08 ± 23.96	63.09 ± 9.47	32.53 ± 5.54	59.26 ± 15.51

Radiolabeled CDV (10 μCi) was added to the cells to a final concentration of 50 μg/ml and 3 days later a methanol extraction was performed. CDV metabolites in the methanol soluble fraction were separated by means of HPLC. Methanol insoluble fractions were digested with NaOH and the amount of CDV incorporated into genomic DNA was measured with a scintillation counter. Mean CDV incorporation and metabolites of at least 4 experiments and SEM are given in pmol per million cells.

A comparison of the sum of all metabolites in tumor cells *versus* normal cells showed higher amounts in PHKs and PET cells than in tumor cell lines (i.e. SiHa, HeLa, C33A, HaCaT, SCC-120 and SCC-147) and HEL cells. HaCaT had the lowest level of total metabolites among all the tested cell types.

**Figure 3:**
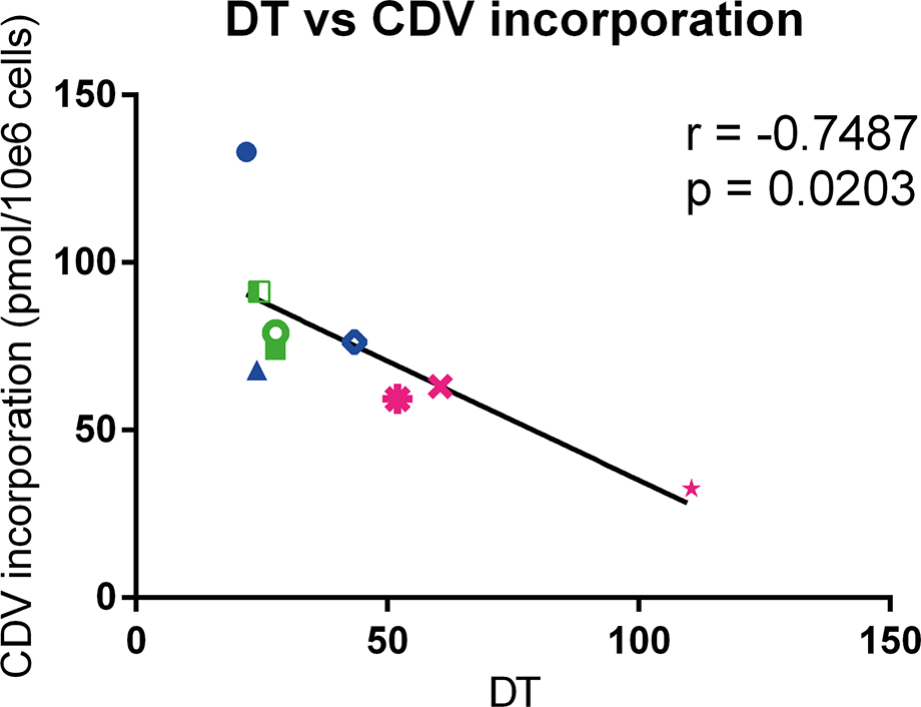
Correlation between doubling time and CDV incorporation at day 3 post infection Pearson correlation was calculated between DT and incorporation of CDV into genomic DNA of the cells. HPV^+^ cell lines are indicated in blue, HPV^−^ in green and the normal cells in pink. Cervical cancer cell lines are indicated by closed symbols and HNSCC by open symbols.

To determine the level of CDV incorporated into DNA, the radioactivity recovered from the methanol insoluble fraction was measured. Normal cells had lower amounts of CDV incorporated into DNA than the tested tumor cells, however the differences were statistically non-significant (Table 2). In Table 3, the incorporation of CDV in tumor cells relative to normal cells is shown. Tumor cells incorporated more CDV than PHKs (1.1 to 2.1 folds), HEL cells (1.1 to 2.2 folds) and PET cells (2.1 to 4.1 folds). For all the tested cell types, including normal cells, a negative correlation (*p* < 0.05) between doubling time and CDV incorporation into DNA was observed at day 3 post-treatment, indicating that fast proliferating cells incorporate more CDV than cells with a slower growth rate (Figure 3).

**Table 3:** CDV incorporation in the tumor cells *versus* normal cells

	Ratio of CDV incorporation between tumor cells and normal cells		
Cell line	PHK	HEL	PET
**SiHa**	2.1	2.2	4.1
**HeLa**	1.1	1.1	2.1
**SCC-147**	1.2	1.3	2.3
**SCC-120**	1.3	1.3	2.4
**C33A**	1.2	1.2	2.3
**HaCaT**	1.4	1.5	2.8

CDV-incorporation in the tumor cells, relative to the normal cells. Tumor cells are shown in the left column and are compared to each of the three normal cells (PHK, HEL and PET).

**Figure 4:**
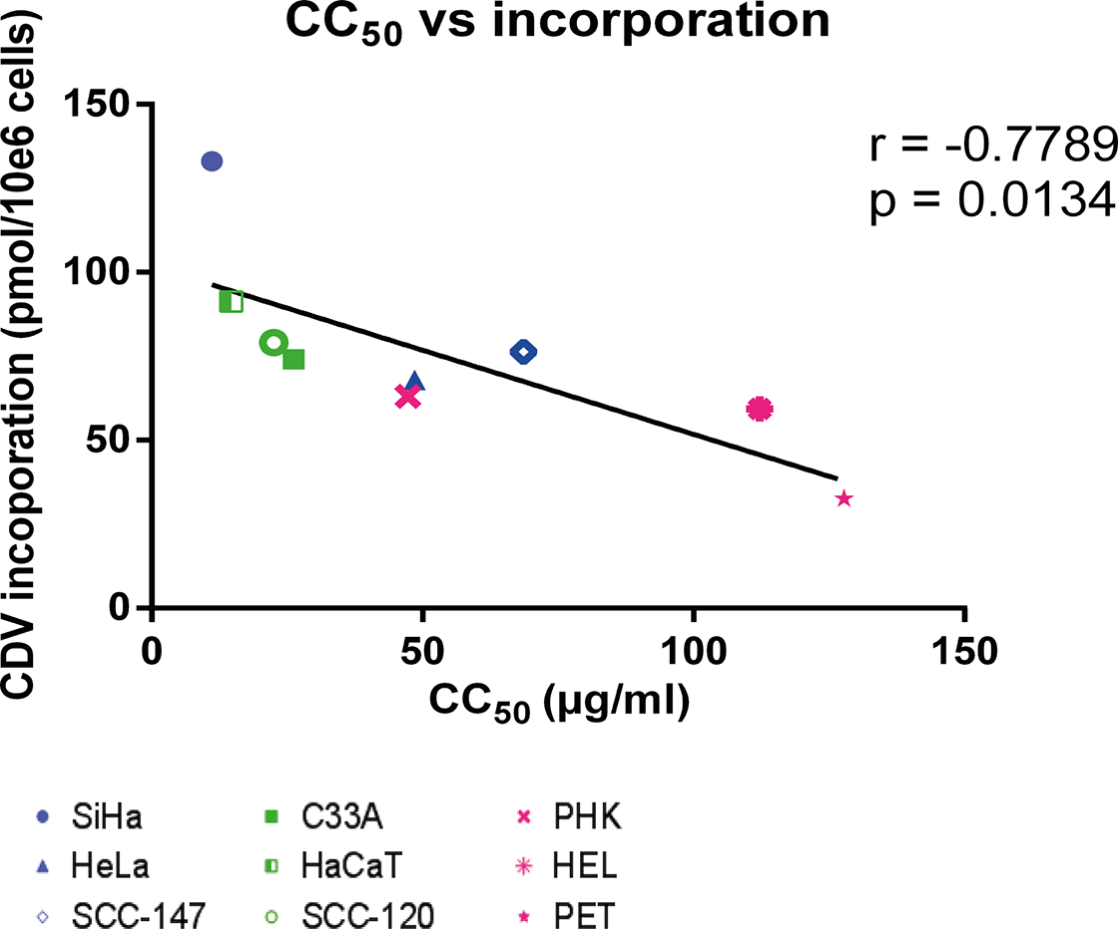
Correlation between CC_50_ values and CDV incorporation at day 3 post-infection Correlation between CC_50_ values and CDV incorporation was evaluated with the Pearson correlation coefficients. A linear regression line was drawn.

We also analyzed whether a correlation between CDV incorporation into genomic DNA and CC_50_ values exists since the latter parameter estimates the net outcome of CDV treatment in terms of antiproliferative effects. A negative correlation (*p* < 0.05) between CDV incorporation and CC_50_'s was observed (Figure 4) revealing that the cells that are more prone to CDV antiproliferative effects (i.e. with low CC_50_ values) had the highest CDV incorporation into the genomic DNA.

### DNA damage signaling after CDV treatment

**Figure 5:**
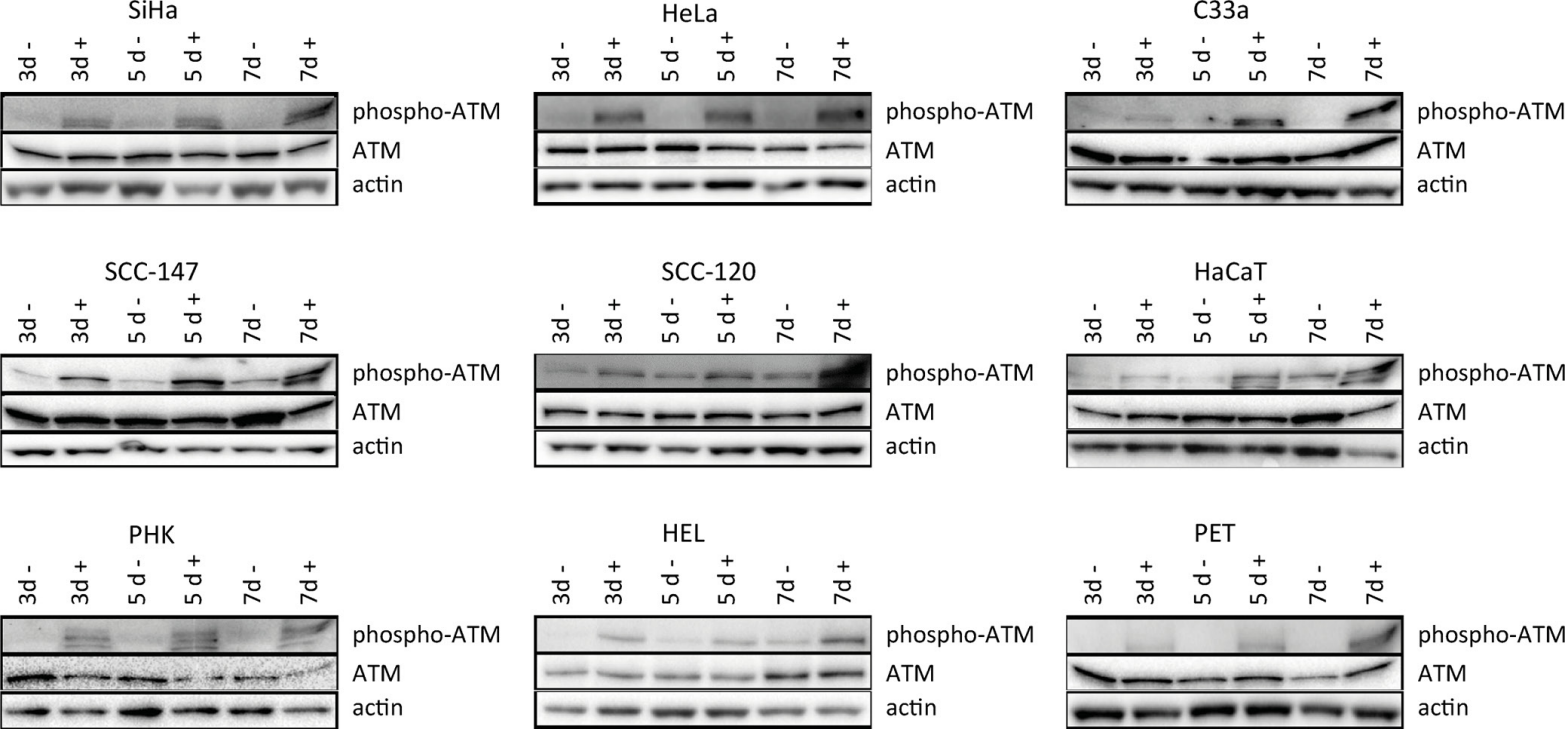
Western blot analysis of DNA damage signaling Protein extracts were obtained at 3, 5 and 7 days of no treatment or treatment with 50 μg/ml CDV. Afterwards, Western blotting was performed. Representative Western blots for each cell line after immunoblotting with ATM and phospho-ATM are shown. Actin was used as a loading control.

**Figure 6:**
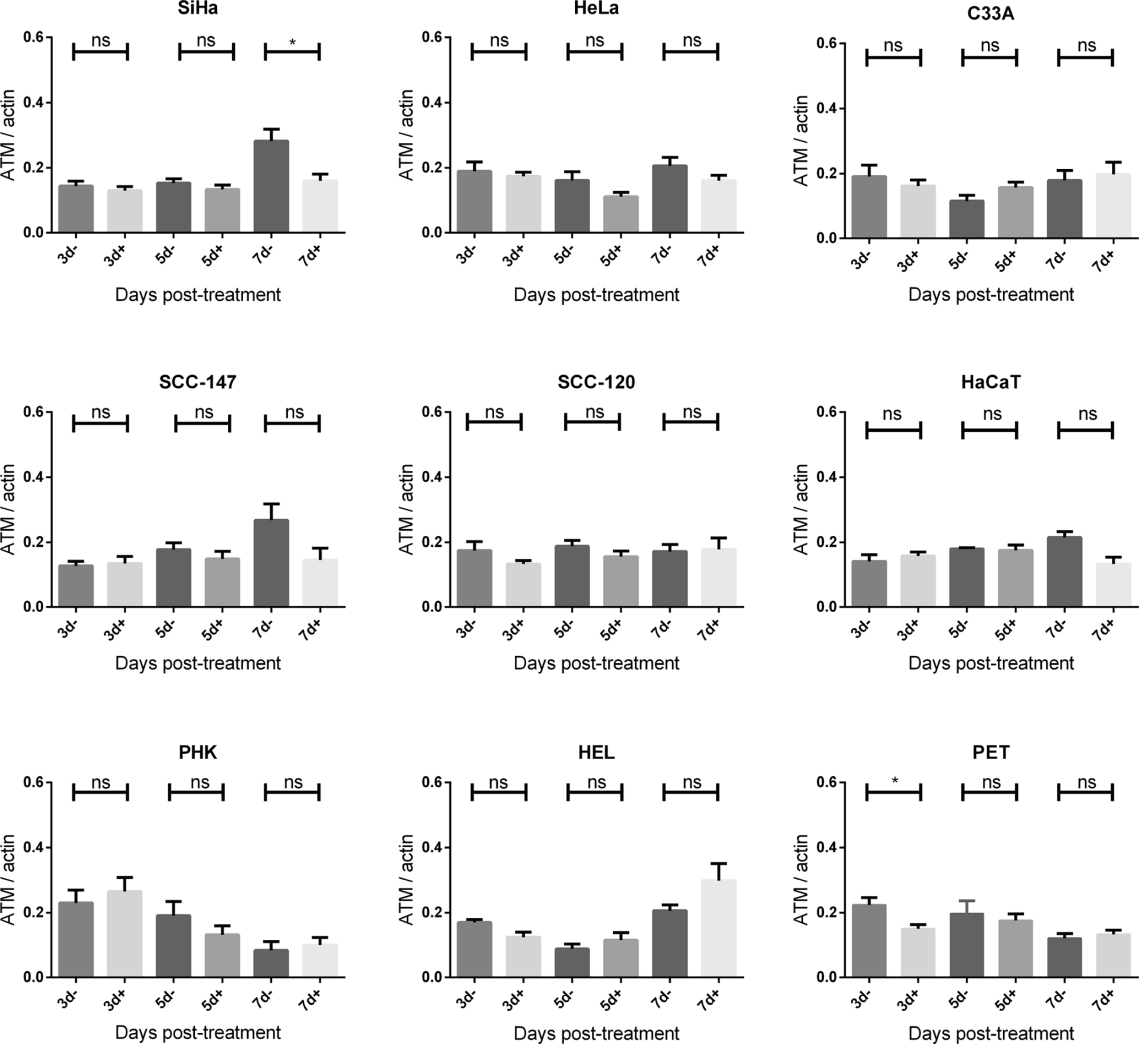
ATM protein expression after CDV exposure Protein extracts were obtained at 3, 5 and 7 days of no treatment or treatment with 50 μg/ml CDV. Afterwards, Western blotting was performed using anti-ATM antibody and anti-actin antibody as a loading control. The amount of ATM relative to actin is shown in arbitrary units (AU) for all the cell lines. For each blot, the sum of intensity values was calculated and used for normalization of the values per blot. Statistical significance was indicated as follows:*p* < 0.05 (*). Values are shown as mean of minimum 3 experiments and SEM.

To investigate DNA damage induction by CDV, the activation of ATM was measured at different time points post-exposure to CDV. Western blot analysis showed that CDV treatment did not increase or decrease the amount of total ATM except for a lower expression of ATM at day 7 in SiHa cells and at day 3 in PET cells (Figures 5 and 6).

**Figure 7:**
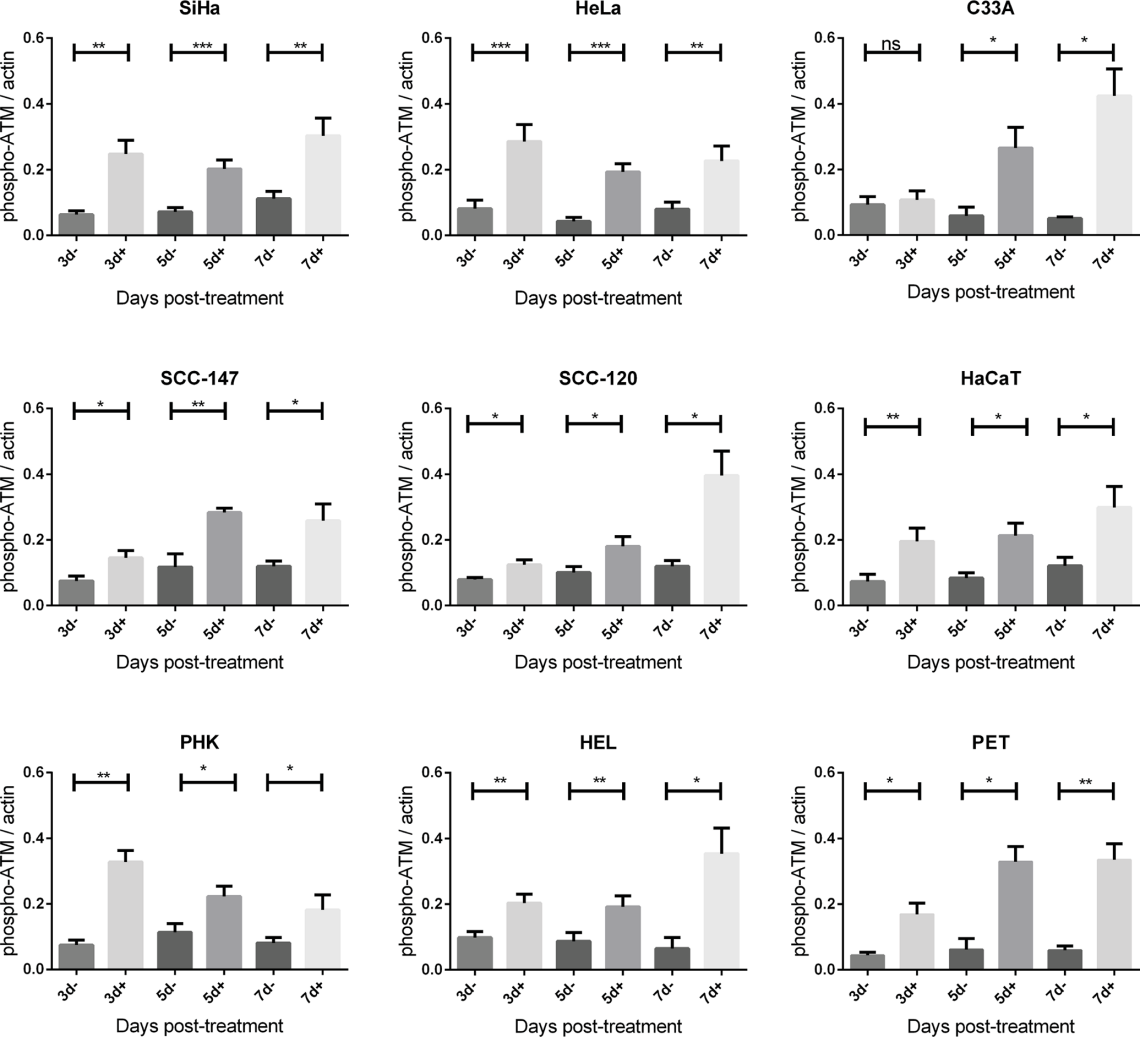
Phospho-ATM protein expression after CDV exposure Protein extracts were obtained at 3, 5, 7 and 10 days of no treatment or treatment with 50 μg/ml. Afterwards, Western blotting was performed using anti-phospho-ATM antibody and anti-actin antibody as a loading control. The amount of phospho-ATM relative to actin is shown in arbitrary units (AU) for all the cell lines. For each blot, the sum of intensity values was calculated and used for normalization of the values per blot. Statistical significance was indicated as follows: *p* < 0.05 (*); *p* < 0.01 (**); *p* < 0.001 (***). Values are shown as mean of minimum 3 experiments and SEM.

Importantly, phospho-ATM (activated ATM) levels were elevated in all CDV treated cells compared to untreated cells at almost all time points (Figures 5 and 7). A significant enhancement of phospho-ATM was seen after 3, 5 and 7 days post-CDV exposure in transformed cell lines (except for C33A in which ATM was not activated after 3 days post-treatment) and in the normal cells (i.e. PHK, HEL and PET).

### DNA damage induced by CDV by means of γ-H2AX analysis

The activation of ATM in CDV treated cells gives a good indication of the induction of DSBs by CDV. However, ATM-activation is not correlated with the amount of DSBs [33]. Therefore, quantification of DSBs was performed using a flow cytometric assay that measures γ-H2AX (i.e. H2AX phosphorylated at S139 by ATM), which is considered a sensitive marker of DSBs.

**Figure 8:**
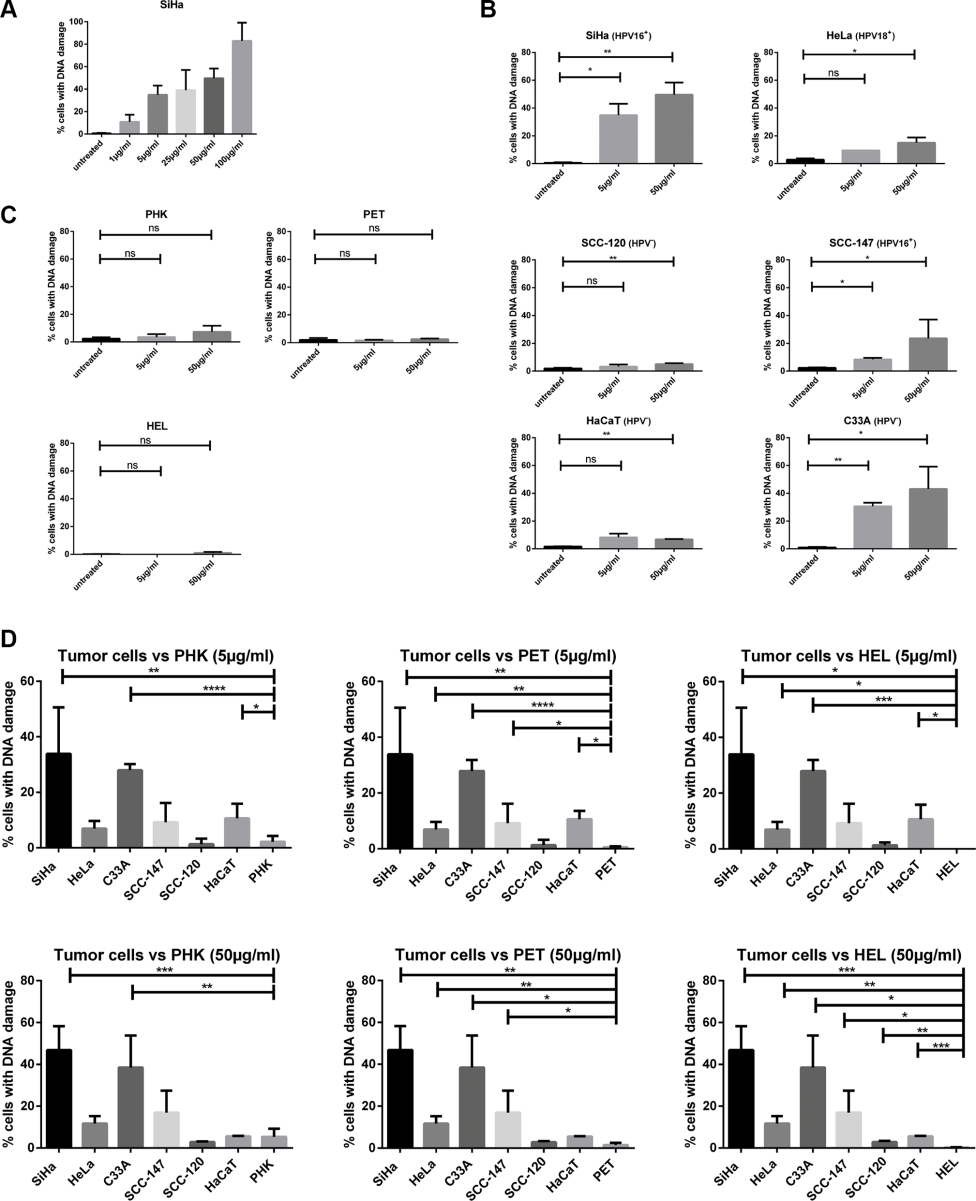
DNA damage induced by CDV by means of γ-H2AX analysis Cells were seeded in culture flasks and 24 h later treated with 5 or 50 μg/ml of CDV. At 3 days post treatment, cells were trypsinized and permeabilized and fixed. Anti-γ-H2AX antibody was used to measure DNA damage by means of flow cytometry. (**A**) DNA damage at increasing CDV concentration in SiHa cells. (**B**) Untreated versus CDV treated tumor cells. (**C**) Untreated versus CDV treated normal cells. (**D**) Comparison of the DNA damage in tumor cell lines and normal cells, 3 days after treatment with 5 μg/ml or 50 μg/ml of CDV. In Figure D, a bar was drawn to indicate only a significant difference between two results. Statistical significance was indicated as follows: *p* < 0.05 (*); *p* < 0.01 (**); *p* < 0.001 (***); *p* < 0.0001 (****). Values are shown as mean of minimum 3 experiments and SEM.

Because SiHa cells had the lowest CC_50_ values among all tested cells, the induction of DSBs by different concentrations of CDV was determined in SiHa cells after 3 days of drug exposure. The percentage of cells with DNA damage increased in function of the CDV concentration (1 μg/ml to 100 μg/ml) (Figure 8A). Based on these data, CDV concentrations of 5 and 50 μg/ml were used to evaluate DNA damage induction in all cell types after 3 days of drug exposure.

When cells were treated with 5 μg/ml CDV, a difference in DNA damage induction between treated and untreated tumor cells was detected for HPV16^+^ cells (SiHa and SCC-147) (*p* < 0.05) and C33A cells (*p* < 0.01) but not for HeLa, SCC-120 and HaCaT cells (Figure 8B). At a CDV concentration of 50μg/ml, a significant difference in DNA damage between treated and untreated cells was observed for the different tumor cell lines tested (Figure 8B). In contrast to tumor cells, normal cells exposed or not to 5 or 50 μg/ml of CDV showed no differences in the percentage of cells with DNA damage (Figure 8C).

A negligible amount of γ-H2AX was found in untreated cells and this intrinsic γ-H2AX can vary among cell lines [34]. Therefore, the percentage of cells with DNA damage in untreated cells was subtracted from that of CDV-treated cells in order to compare the different cell types. The comparison of CDV-induced DNA damage in tumor cell lines *versus* normal cells is shown in Figure 8D. SiHa, C33A and HaCaT (in contrast to the other tumor cells) showed higher amounts of DNA damage than PHKs after 5 μg/ml CDV treatment (Figure 8D, up). All tumor cells, except for SCC-120, experienced more DNA damage than PET cells upon treatment with 5μg/ml CDV. In addition, at this dose of CDV, HEL cells underwent significantly less DNA damage than most tumor cells, except for SCC-120 and SCC-147. When the DNA damage in normal *versus* tumor cells after treatment with 50 μg/ml of CDV was examined (Figure 8D, down), all tumor cells lines showed significantly higher DNA damage than HEL and PET cells. Only SiHa and C33A cells underwent significantly more DNA damage than PHKs at the highest CDV concentration tested. (except for SCC-120 and HaCaT)

Flow cytometric analysis using propidium iodide (PI) and γ-H2AX staining showed that CDV-induced DSBs were present in each phase of the cell cycle (Data not shown). Thus, tumor cells in all phases of the cell cycle were equally sensitive to DNA damage.

**Figure 9:**
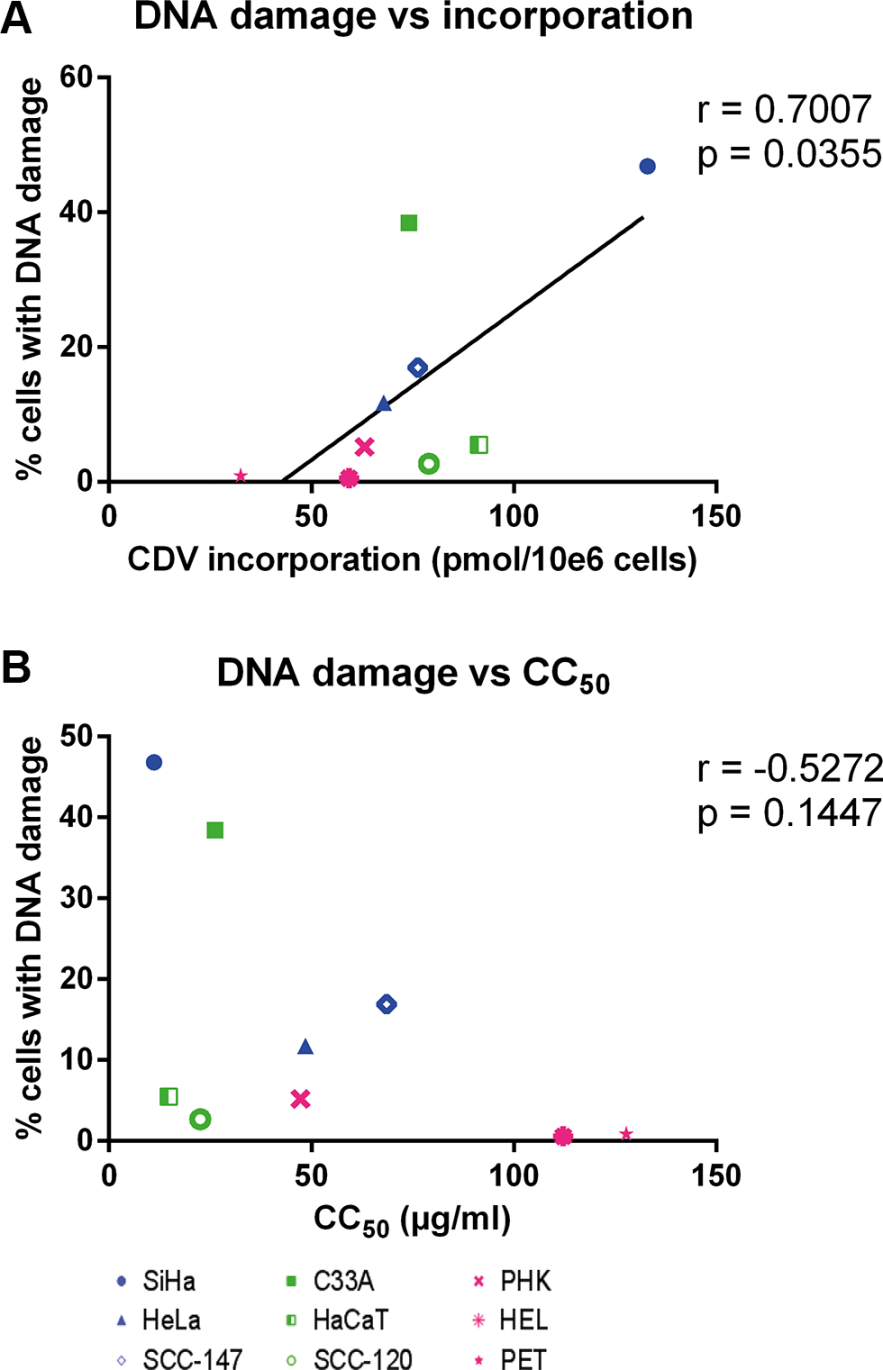
Correlation between CC_50_ values, DNA damage and CDV incorporation at day 3 post-treatment Correlations between (**A**) DNA damage and CDV incorporation and (**B**) CC_50_ values and DNA damage were evaluated with the Pearson correlation coefficients. A linear regression line was drawn when a significant correlation was present.

Finally, the CC_50_ values and the levels of CDV incorporation and drug-induced DNA damage were compared to detect potential correlations. A positive correlation (*p* < 0.05) between CDV incorporation into DNA and DNA damage was found (Figure 9A) while there was no correlation between CC_50_ values and DNA damage (Figure 9B).

## DISCUSSION

The antiproliferative effects of CDV were studied in nine HPV^+^, six HPV^−^ cell lines (including cervical carcinoma and HNSCC) and three different normal cell types (PHKs, HEL and PET cells). Tumor cells proved to be more responsive to CDV antiproliferative activities than normal cells, as indicated by the lower CC_50_ values obtained for the tumor cell lines. Here we have demonstrated that CDV is also active against HNSCC regardless of HPV status. The growth of PHKs was relatively more inhibited by CDV in comparison with the two other tested normal cells (HEL and PET), which could be explained by the fact that keratinocytes were artificially grown in a monolayer causing stress to these cells that are programmed to differentiate.

Our results show that the antiproliferative activity of CDV is time- and dose-dependent, as shown previously [23]. Interestingly, a decrease in CC_50_ values in function of time was not found for other anti-tumor drugs such as cytarabine and 5-fluorouracil [23]. Although differences in response to CDV were detected among the various cell lines, these differences were not related to HPV status nor to tumor type (i.e. originating form cervical or head and neck tissues), indicating that CDV may be considered a broad-spectrum anti-tumor agent. Hadaczek *et al.* also showed that CDV inhibits glioblastoma both *in vitro* and *in vivo*, regardless of the presence of human cytomegalovirus (HCMV) [25], however a role for HCMV in the pathogenesis of glioblastoma is controversial [35]. It was previously shown that the *in vitro* and *in vivo* antiproliferative activities of CDV against nasopharyngeal carcinoma (which is driven by Epstein-Barr virus) were not due to inhibition of the viral DNA polymerase [27]. Furthermore, the activity of CDV against polyomavirus (PyV)-induced hemangioma *in vivo* could not be explained by an antiviral mechanism [26].

The correlation between CC_50_ values and doubling time indicates that the selectivity of CDV may be explained, at least in part, by a more potent inhibition of cell growth on fast proliferating cells (tumor cells) than on normal cells (which have slower proliferation rates). When the normal cells were excluded from the analysis, a significant correlation between CC_50_'s and doubling time was only observed at day 3 post-treatment but not at later time points. Spanos *et al.* described a correlation between CDV antiproliferative activities and doubling time in HNSCC after 4 days of treatment [36].

CDV selectivity for tumor cells could also be explained by a differential metabolism of the drug in malignant *versus* normal cells [37]. In all the cell types tested here, unmetabolized CDV and CDVp-choline were the most abundant fractions detected, what is in agreement with previous data on CDV metabolism [32]. Our findings indicate that a substantial amount of the administered CDV is not metabolized. Furthermore, the long half-life (87 h) of CDVp-choline [38] could explain its high abundance in the cells. The monophosphate form (CDVp), which has the shortest half-life (24 h), was the least abundant metabolite. Among the cell lines evaluated, HaCaT displayed a significantly lower sum of CDV metabolites, which could be attributed to changes in uptake and/or efflux of CDV, rather than to a difference in drug metabolism, since the amount of parent CDV was also lower in HaCaT than in the other cells. The higher levels of total metabolites in normal cells (PHK and PET) may be due to a higher activity of the enzymes responsible for CDV activation and/or more efficient drug uptake by these cells. However, CDV incorporation into DNA was lower in normal cells than in transformed cells. One can hypothesize that normal cells are able to remove incorporated CDVpp while transformed cells have alterations in their DNA damage repair mechanisms impeding drug excision. On the other hand, tumor cells proliferate faster than normal cells and therefore can incorporate more CDVpp. This last hypothesis is supported by the correlation between doubling time and CDV incorporation into DNA.

Induction of DNA damage may be an additional factor in the selectivity of CDV for tumor cells since normal cells are capable of repairing DNA damage while tumor cells often lack this capability [7]. The E6 and E7 HPV oncoproteins override the G1/S-phase checkpoint hampering DNA damage repair in HPV^+^ transformed cells [5, 6]. Previous publications also suggested that HPV^+^ cells are more sensitive to radiation due to an impaired DNA damage response [39, 40]. Our experiments show that the failure of HPV^+^ cells to repair DNA damage does not occur at the beginning of the homologous recombination repair pathway since increase in phospho-ATM after CDV treatment was found in all HPV^+^ cells. The augmentation of phospho-ATM in CDV-treated cells is in line with a previous gene expression analysis of PHKs [32], suggesting that induction of DSBs and activation of DNA DSB repair by homologous recombination occurs following CDV treatment.

Because phospho-ATM levels were elevated in all cell lines exposed to CDV (including normal cells), DSBs were quantified by measuring γ-H2AX. Importantly, in tumor cells there was significantly more DNA damage in CDV-treated than in untreated cells, which was not the case in normal cells. Furthermore, a statistically significant difference in induction of DNA damage was seen between most of the tumor cell lines and normal cells, as evidenced by γ-H2AX levels. Induction of DSBs by CDV was not linked to HPV positivity nor tumor type (cervical or head and neck), similar to CDV antiproliferative effects and drug incorporation into DNA. Nevertheless, no correlation between DNA damage and CDV antiproliferative effects were observed suggesting that the mode of action of CDV cannot be solely explained by induction of DNA damage.

Regarding CDV antiproliferative effects, SiHa cells (HPV16^+^ cervical carcinoma) proved to be the most sensitive among all the tumor cell lines evaluated in this study. Even though H2AX phosphorylation in CDV-exposed SiHa and C33A cells was comparable, C33A showed higher CC_50_ values than SiHa. HeLa cells presented higher CC_50_ values than SCC-120 and HaCaT cells, but had more DNA damage after CDV exposure than SCC-120 and HaCaT cells. One can hypothesize that induction of DNA damage would lead to a decrease in CC_50_ values, unless the DNA damage is repaired. When the two tumor cell lines showing the lowest DNA damage (i.e. HaCaT and SCC-120) were excluded from the analysis of CC_50_'s and DNA damage, a significant correlation (*p* < 0.05) between DNA damage and antiproliferative effects was found (Data not shown). These data suggest that HaCaT and SCC-120 cells are capable of (partially) repairing DNA damage although these cells were also shown to be sensitive to CDV. Taken together, our findings indicate that CDV antiproliferative activities are due to the induction of DNA damage but in contrast to most chemotherapeutics, additional effects can be deduced. Induction of apoptosis, cell cycle arrest or an increase in tumor suppressors such as p53, pRb or p21, which have been already described for CDV-treated HPV^+^ cells, could be a consequence of CDV incorporation into DNA [41]. However, activation of inflammatory responses (such as “NF-κB signaling” and “acute phase response”), retinoid X receptor pathways and Rho GTPase pathways among others should be taken into account to explain CDV antiproliferative activities [32].

One would expect no ATM phosphorylation in normal cells since there is no phosphorylation of H2AX in normal cells following 3 days of CDV treatment. However, in all the normal cells (PHK, HEL and PET) a significant enhancement of phospho-ATM was observed after 3, 5 and 7 days of CDV treatment. This discordance may be explained by the fact that ATM is not used as a marker for DSBs because it is not correlated with the amount of DSBs (ATM only needs 8 breaks per cell in order to be phosphorylated) [42]. In contrast, the intensity of γ-H2AX immunofluorescence was shown to correlate with the frequency of DSBs [34]. For this reason γ-H2AX was used in our studies as a quantitative marker for DSBs. We assume that CDV causes DSBs in normal cells that are sensed by ATM and then repaired by DNA repair mechanisms. Even a small amount of DSBs would be sufficient to activate ATM but it would not be high enough to cause a significant difference in phosphorylation of H2AX. After an initial step of ATM phosphorylation, the existing damage is evaluated [43] and if the damage is repaired, phospho-ATM is dephosphorylated by Wip1 and PP2A. In case of persistent DNA damage, there is a new peak of ATM-phosphorylation [9, 43]. Thus, in contrast to normal cells, phospho-ATM may accumulate in tumor cells after CDV exposure because of persistent DNA damage. The accumulation of breaks in tumor cells is deduced from the high amount of cells with γ-H2AX immunofluorescence. To conclude, the levels of γ-H2AX and phospho-ATM do not evolve in parallel because γ-H2AX is correlated with the amount of DSBs whereas activation of ATM takes place even when there is only a limited amount of DSBs and does not correlate with the amount of DSBs [33].

A discrepancy between γ-H2AX and phospho-ATM was also observed for C33A cells that showed a high amount of phosphorylated H2AX upon 3 days of 50 μg/ml CDV treatment while no significant increase of phospho-ATM was observed at this time point. This could be explained by the fact that in response to DNA damage, all three phosphatidylinositol 3-kinase-related kinases (PIKKs): ATM, ataxia telangiectasia and Rad3-related protein (ATR), and DNA-dependent protein kinases (DNA-PKcs) can phosphorylate H2AX as previously reported following DNA damage caused by ionizing radiation [44]. Additionally, it is not yet clear which PIKK is responsible for H2AX phosphorylation during apoptosis. This is consistent with formerly reported data showing that UV-induced replication stress triggers activation of ATR and DNA PKcs rather than ATM [45]. Most publications, however, point out that the induction of DSBs triggers phosphorylation of H2AX by ATM [33, 46, 47].

In conclusion, we have shown here the antiproliferative effects of CDV against cervical carcinoma and HNSCC HPV^+^ and HPV^−^ cells. CDV antiproliferative effects were correlated with drug incorporation into DNA. Moreover, CDV incorporation was correlated with DNA damage, indicating that CDV incorporation causes DNA damage. However, the anti-tumor effect of CDV cannot be explained exclusively by DNA damage, since no correlation was observed between DNA damage and CDV antiproliferative effects. These data support the concept of CDV as a general anti-cancer agent not restricted to HPV^+^ tumor cells [25–28, 48]. Our results contribute to a better insight into the selective mechanism of CDV anti-tumor activities which is crucial for the development of new strategies to treat HPV-associated neoplasias that cannot be cured with standard therapies. In addition, our work sets up the basis for further investigations on the mode of action of CDV and on the DNA repair mechanism.

## MATERIALS AND METHODS

### Cells

HPV^+^ and HPV^−^ cell lines derived from cervical carcinoma or head and neck squamous cell carcinoma (HNSCC) were compared to spontaneously transformed keratinocytes (HaCaT), primary human keratinocytes (PHKs), primary epithelial tonsil (PET) cells and human embryonic lung (HEL) fibroblasts. Cervical cancer cell lines: SiHa (HPV16^+^) (ATCC HTB-35™), HeLa (HPV18^+^) (ATCC CCL-2™), Caski (HPV16^+^) (ATCC CRL-1550) and C33A (HPV^−^) (ATCC HTB-31™) were obtained from ATCC (Manassas, USA). CK1 (HPV33^+^) cells were kindly provided by Jaques Piette (Departement of Life Sciences, University of Liège, Belgium). The HNSCC SCC-9 (ATCC CRL-1629^™^) and SCC-4 (ATCC CRL-1624™) (both HPV^−^) were obtained from ATCC; 93VU147T later called SCC-147 (HPV16^+^) and 93VU120 later called SCC-120 (HPV^−^) were kindly provided by Mario Hermsen (Department of Otolaryngology, Instituto Universitario de Oncología del Principado de Asturias (IUOPA), Spain); UM-SCC-47 (HPV16^+^), UM-SCC-104 (HPV16^+^) and UM-SCC-38 (HPV^−^) were purchased from the University of Michigan (USA); UT-SCC-45 (HPV33^+^) and UD-SCC-2 (HPV16^+^) were kindly provided by Reidar Grénman (Dept. of Otorhinolaryngology-Head and Neck Surgery Turku University Central Hospital, Finland) and Prof. Thomas Hoffman (Department of Otorhinolaryngology, University hospital Ulm, Baden-Württemberg), respectively. HPV^−^, *in vitro* spontaneously transformed keratinocytes from histologically normal skin (HaCaT) were kindly provided by F. De Marco (Laboratory of Virology, Regina Elena Institute for Cancer Research, Rome, Italy). Human embryonic lung (HEL) fibroblasts (HEL-299: ATCC CCL-137) were grown in Earle's minimum essential medium (MEM Earle's, Life Technologies, Merelbeke, Belgium) containing 8% fetal calf serum. PET and PHK were isolated from tonsils and from neonatal foreskins, respectively, as previously described [49] and cultured in Keratinocyte-SFM Medium (Gibco, Life Technologies). All cancer cell lines were maintained in Dulbecco's modified Eagle's medium (Gibco, Life Technologies) except for SCC-4 and SCC-9 which were grown in Dulbecco's modified Eagle's medium plus F12 (Gibco, Life Technologies). All media were supplemented with 1% non-essential amino acids (MEM NEAA, Gibco by Life Technologies), 1% sodium pyruvate 100 mM (Gibco by Life Technologies), 1% Penicillin/Streptomycin/Glutamine 100× (Gibco by Life Technologies) and 1% HEPES 1M (Gibco by Life Technologies).

### Presence of HPV genome in the different cell lines

The presence or absence of the HPV genome in all the aforementioned cells was confirmed by performing a specific PCR. DNA was extracted with a QIAamp DNA mini kit (Qiagen Benelux, Netherlands) following the instructions of the manufacturer, followed by PCR to confirm whether the cell lines were HPV16^+^, HPV18^+^, HPV33^+^ or HPV^−^. Specific primers for each HPV type (Supplementary Table 1) were purchased from Eurofins (Brugge, Belgium).

### Antiviral compound

Cidofovir (CDV) or (S)-HPMPC, [(S)-1-[3-hydroxy-2-(phosphonomethoxy)propyl]cytosine] was obtained from Gilead Sciences (Foster city, Ca, USA). It was prepared in PBS at a stock concentration of 10 mg/ml. Radiolabeled [5-^3^H]-CDV (1mCi/ml; specific activity: 25 Ci/mmol) was purchased from Moravek Biochemicals (Brea, CA, USA) and stored at −20°C in ethanol/water 1:1.

### Antiproliferative effects

Cells were seeded in 96-well microtiter plates at an amount ranging from 2.5 × 10^3^ to 6 × 10^3^ cells per well, determined for each cell line (2.5 × 10^3^ for SiHa, HeLa, C33A, SCC-120, SCC-9, UM-SCC-47 and UM-SCC-38; 3 × 10^3^ for HaCaT; 3.5 × 10^3^ for HEL; 4 × 10^3^ for Caski, SCC-4, CK1, UT-SCC-45 and UD-SCC-2; and 6 × 10^3^ for SCC-147, PHK, PET and UM-SCC-104). After 24 hours, medium containing different concentrations of CDV, ranging from 0.05 μg/ml to 200 μg/ml, was added to the cells (in duplicate). After 3, 5, 7 and 10 days, the cells were trypsinized and counted with a Coulter counter. The antiproliferative effects were expressed as the compound concentration needed to inhibit cell growth by 50% (CC_50_).

### *In vitro* growth rate

The doubling time (DT) of the different cell lines was determined in 96-well microtiter plates. At several time points, the number of cells was determined with a Coulter counter. DT was calculated with the formula: DT = (t_2_-t_1_)/(log_2_N_2_-log_2_N_1_), where t_1_ and t_2_ are the times (in hours) at which the cells were counted, and N_1_ and N_2_ are the cell numbers at times t_1_ and t_2_.

### Metabolism and incorporation of CDV into DNA

Cells were grown in 75 cm^2^ culture flasks for 24 hours before adding unlabeled CDV combined with [5-^3^H]-CDV at a final concentration of 50 μg/ml and 10 μCi per flask. After 3 days of incubation, samples for HPLC analysis were prepared by methanol extraction as described previously [32]. The soluble fraction of this MeOH extraction (200 μl) was injected onto a HPLC system equipped with an anion-exchange Partisphere SAX column (Hichrom limited, Berkshire, UK) and separation was performed with a gradient of two phosphate buffers; 5 mM NH_4_H_2_PO_4_, pH 6.5 and 0.3 M NH_4_H_2_PO_4_, pH 6.5 at a flow rate of 2 ml/min. One-minute fractions of the eluate were collected, mixed with HiSafe 3 cocktail (Perkin Elmer, Massachusetts, USA) and analyzed for radioactivity in a scintillation counter. The retention times of CDV and its metabolites were: 3 min for CDVp-choline, 5 min for CDV, 15 min for CDVp, and 19 min for CDVpp. To determine the incorporation of CDV into nucleic acids, the methanol-insoluble pellets were digested with 500 μl 5N NaOH during 24 hours at 37°C. The solution was neutralized with 500 μl 5N HCl, then mixed with HiSafe 3 cocktail and analyzed for total radioactivity.

### Protein extract preparation and immunoblotting

Protein extracts were obtained at 3, 5 and 7 days of no treatment or treatment with 50 μg/ml CDV. Whole cell lysates were prepared in Ripa buffer (Thermo Scientific, Brussels, Belgium) containing protease (Complete Mini, EDTA-free, Sigma-Aldrich, Diegem, Belgium) and phosphatase inhibitors (Active Motif, La Hulpe, Belgium). Protein concentrations were measured by the BCA assay (Thermo Scientific). Separation of proteins was performed with SDS-PAGE. Western Blot analysis was performed with anti-phospho-ATM (Ser1981) clone 10H11.E12 (Millipore, Billerica, MA, USA) and anti-ATM antibody (ab32420, Abcam, Cambridge, UK). Relative quantification was performed using actin (ab3280, Abcam) as a loading control. For each blot, the sum of intensity values was calculated and used for normalization of the values per blot.

### DNA damage induced by CDV by means of γ-H2AX analysis

DNA damage was evaluated by means of a FlowCellect TM cell cycle Checkpoint H2A.X DNA damage kit (Millipore). Briefly, cells were seeded in culture flasks for 24 hours before adding 5 or 50 μg/ml CDV. After three days of incubation, the cells were fixed, permeabilized and stained with propidium iodide and anti-γ-H2AX antibody. Samples were read with a BD FACSCalibur.

### Statistical analysis

Statistical analyses were performed with GraphPad Prism 6 software (GraphPad Software Inc., La Jolla, CA, USA). To evaluate the antiproliferative effects of CDV over time and to compare treated cells with untreated cells for DNA damage, a paired *t*-test was used. Unpaired *t*-test was used to compare different cell lines for CC_50_ values, DT, metabolites, incorporation into DNA, and DNA damage. Pearson correlations were assessed comparing DT and CC_50_; CC_50_ and DNA damage; CC_50_ and DNA incorporation and DNA damage and DNA incorporation. *P* < 0.05 was considered as statistically significant.

## SUPPLEMENTARY MATERIALS


