# Supplementary material for: Cidofovir is active against human papillomavirus positive and negative head and neck and cervical tumor cells by causing DNA damage as one of its working mechanisms
Source: Oncotarget. 2016 Jun 16;7(30):47302–18. doi: 10.18632/oncotarget.10100 (PMC5216943; doi:10.18632/oncotarget.10100)
Supplement: Supplementary file 1 [file oncotarget-07-47302-s001.pdf]

Cidofovir is active against human papillomavirus positive and negative head and neck and cervical tumor cells by causing DNA damage as one of its working mechanisms

Supplementary Materials

Supplementary Table S1: Primers used for HPV typing by means of PCR

Primers used for PCR			
HPV type	E6/E7 primer	sequence (5′–3′)	amplicon length (bp)
HPV 16	forward	GTG GAC CGG TCG ATG TAT GTC T	209
	reverse	TCC GGT TCT GCT TGT CCA GC	
HPV 18	forward	AGT GCC ATT CGT GCT GCA AC	98
	reverse	ATG TTG CCT TAG GTC CAT GCA T	
HPV 33	forward	AAT ATT TCG GGT CGT TGG GC	109
	reverse	ACC GTT GGC TTG TGT CCT CTC A	

HPV typing was performed using specific forward and reverse primers for E6/E7 of HPV 16, 18 and 33.
